# Prediction of the Secondary Arms Spacing Based on Dendrite Tip Kinetics and Cooling Rate

**DOI:** 10.3390/ma17040865

**Published:** 2024-02-13

**Authors:** Ibrahim Sari, Mahmoud Ahmadein, Sabbah Ataya, Lakhdar Hachani, Kader Zaidat, Nashmi Alrasheedi, Menghuai Wu, Abdellah Kharicha

**Affiliations:** 1 Metallurgy Department, Montanuniversitaet of Leoben, Franz-Josef-Str. 18, A-8700 Leoben, Austria; 2 Department of Production Engineering and Mechanical Design, Tanta University, Tanta 31512, Egypt; 3 Department of Mechanical Engineering, Imam Mohammad Ibn Saud Islamic University, Riyadh 11564, Saudi Arabia; 4 Laboratoire Physique des Matériaux, Université Amar Telidji-Laghouat, Route de Ghardaia, Laghouat BP 37G, Algeria; 5 University Grenoble Alpes, Grenoble-INP, CNRS, SIMaP, F-38000 Grenoble, France

**Keywords:** secondary dendrite arm spacing, directional solidification, sub-grid model, microstructures

## Abstract

Secondary dendrite arm spacing (SDAS) is one of the most important factors affecting macrosegregation and mechanical properties in solidification processes. Predicting SDAS is one of the major parameters in foundry technology. In order to predict the evolution of microstructures during the solidification process, we proposed a simple model which predicted the secondary dendrite arm spacing based solely on the tip velocity (related to the tip supersaturation) and cooling rate. The model consisted of a growing cylinder inside a liquid cylindrical envelope. Two important hypotheses were made: (1) Initially the cylinder radius was assumed to equal the dendrite tip radius and (2) the cylindrical envelope had a fixed radius in the order of the dendrite tip diffusion length. The numerical model was tested against experiments using various Pb–Sn alloys for a fixed temperature gradient. The results were found to be in excellent agreement with experimental measurements in terms of SDAS and dendrite tip velocity prediction. This simple model is naturally destined to be implemented as a sub-grid model in volume-averaging models to predict the local microstructure, which in turn directly controls the mushy zone permeability and macrosegregation phenomena.

## 1. Introduction

Solidification is one of the most significant phenomena for many materials, particularly metals and alloys. Researchers have made significant progress in our understanding of solidification microstructures over the past decades. During the solidification process, the solid–liquid interface becomes unstable due to the thermal or solute gradients. These interface perturbations indicate the formation of different solid structures such as cells and dendrites or both at the same time where the cell-to-dendrite transition (CDT) occurs. The dendritic microstructure is characterized by an array of primary dendrite arm spacings, *λ*_1_, secondary dendrite arm spacings, *λ*_2_, and even higher-order arms that form a complex structure [1]. Due to the importance of predicting secondary arm spacing in foundry technology, numerous studies of solidified binary alloy microstructures have been performed to determine experimentally the interdependent structure parameters such as *λ*_1_ and *λ*_2_, and solidification parameters, such as temperature gradient (*DT*), growth rate (*V*), and cooling rate (*CR*). In directional solidification studies, the growth velocity, V, and the temperature gradient in the liquid, DT, may be controlled independently, allowing one to investigate the dependence of structural parameters (*λ*_1_, *λ*_2_) on either *DT* (at constant *V*) or *V* (at constant *DT*). Several researchers [1,2,3,4,5] report that as the growth rate, temperature gradients, and cooling rate increase, the primary and secondary dendritic arm spacing decrease. Roósz et al. [6] measured the secondary dendrite arm spacing (SDAS) of an Al-7 wt% Si alloy at various locations within solidified samples, subjected to different temperature conditions, using microgravity experiments. By considering the actual liquidus temperature (T_L_) affected by macrosegregation, SDAS was calculated as a function of average processing parameters and the actual liquidus temperature was calculated using the classical Kirkwood’s equation. These studies demonstrated that the predicted SDAS is dependent on the temperature range and various solidification parameters, such as front velocity and temperature gradient. The authors reported that the experimental and the predicted SDAS were in good agreement. Roósz et al. [7] calculated the SDAS based on two methods. The first was the empirical method, which is known to use the local solidification time, which can be derived either from measured cooling curves (equiaxed solidification), or calculated from the temperature gradient and front velocity (directional solidification). This equation is not usable for calculating the SDAS during solidification (it is possible to calculate the final SDAS in an arbitrary position of the sample). The second method is known as a dynamical method; the SDAS can be calculated at the end and during solidification. The results of the two simulation methods were compared with the results of unidirectional solidification experiments performed with an Al-7% Si alloy. Comparing the two calculation methods, the authors stated that the correctness of the methods was similar. However, the simulation results in terms of microsegregation will be more correct using the dynamical method. Their study demonstrated that using the dynamical method based on the known cooling curve, the SDAS can be calculated at the end and during solidification in good agreement with these experimental results. Furthermore, the research reveals a correlation between the final SDAS and the cooling rate: higher cooling rates produce a finer SDAS, while a longer local solidification time leads to the formation of coarser SDAS. Nikolić et al. [8] built a deep learning (D_L_) model for SDAS prediction through a high-pressure die-cast of an EN AC 42,000 AlSi7Mg alloy. The authors predicted the SDAS based on processing parameters: pouring temperature, insulation on the riser, and chill-specific heat, while the dataset was based on numerical simulation results. The authors claimed that the technique based on DL can predict SDAS with very high accuracy. Zhang et al. [9] used the CAFE model of ProCAST to predict the solidification process of different slabs of steel alloys. Based on the simulation of the slab temperature field, a secondary dendrite arm spacing model was established. Their research focused on investigating the relationship between element content, secondary dendrite arm spacing (SDAS), equiaxed crystal ratio (ECR), and macrosegregation in continuously cast experimental slabs. The authors reported that macrosegregation was closely related to the SDAS. The SDAS increased with increasing C and Si content. Also, smaller SDAS could make the solidification structure more compact. Ferreira et al. [10] have used reliable methods to correlate the thermal parameters with secondary dendrite arm spacing during unidirectional solidification of an Al-4.5 wt%Cu alloy. Indeed, spacing of the arms for the studied alloy was numerically predicted using a phase-field model and validated against the experiments. The authors observed that the secondary dendrite arm spacing decreased as the cooling rate increased. Ramirez-Vidaurri et al. [11] have studied the solidification evolution of an ASTM F75, [12] alloy during directional solidification. Indeed, their work was devoted to obtain SDAS–time and SDAS–cooling rate relationships. They found coarser SDAS was obtained with a slower cooling rate. Also, the authors proposed a model based on a lateral-remelting mechanism to describe the SDAS coarsening of multicomponent alloys. The model satisfactorily reproduced the experimental results. Üstün and Çadirli [13] have studied the effect of growth rate on coarsening of secondary dendrite arm spacings in directional solidification of an Al-8.8La-1.2Ni ternary alloy. They found that the secondary dendritic arm spacing was very sensitive to growth rate and an increase in growth rate from 8.3 to 83 μm/s decreased SDAS from 60 to 25 μm. Cicutti et al. [14] developed a simple mathematical model able to estimate the relationship between primary and secondary dendrite arm spacing in continuous casting products for low-carbon steels. The model predicted a λ_1_/λ_2_ ratio of approximately 2.6 which was almost constant along the slab thickness. The results were found to be in good agreement with published experimental data. Ren et al. [15] have developed a comprehensive numerical model using the finite volume method that accurately predicts the occurrence of solute enrichment-induced dendritic fragmentation in directional solidification of nickel-based superalloys. Their model revealed a remarkable interplay between temperature gradient, cooling rate, and dendrite arm spacing in controlling dendritic fragmentation. Under a higher temperature gradient, the primary dendrite tip experienced direct melting by the solute-enriched melt, preventing fragment formation during the re-melting process. In contrast, a relatively low-temperature gradient promoted larger dendritic arm spacing, providing ample space for melt convection. This facilitated the dilution of solute in the interdendritic region by the peripheral melt, effectively inhibiting further solute enrichment and dendritic fragmentation. The authors also claimed that for low cooling rates, dendritic fragmentation was not observed. However, a higher cooling rate promoted the growth of ternary dendritic arms on the side branches, hindering the transfer of solute-enriched melt to the growing dendrite. This reduced solute transfer led to a lower segregation index in the channel and facilitated channel blockage. The model successfully captured these intricate relationships between temperature gradient, cooling rate, solute transport, and dendritic fragmentation, demonstrating a good agreement with experimental observations. In a remarkable study, Ren et al. [16] proposed a novel model to simulate the dynamic evolution of dendrite morphology during both growth and remelting processes. The model was successfully applied to simulate the growth of a single Al-4.5 wt% Cu alloy nucleus immersed in an undercooled melt. Their simulations revealed a distinct fragmentation mechanism, where fragment formation occurred primarily at the connection points between downstream dendrite arms and vertical primary dendrite arms, while no fragment formation was observed on the upstream side. This phenomenon was attributed to the preferential melting of downstream arms due to their larger interdendritic spacing and increased solute enrichment. Additionally, the researchers observed that the presence of forced convection significantly enhanced the growth rate of upstream dendrite arms, further contributing to the preferential fragmentation of downstream arms. Notably, the model’s predictions for dendrite tip velocity were found to be in reasonable agreement with analytical results for different undercooling conditions. Zheng et al. [17] used a phase-field model to simulate the evolution of dendritic structure during solidification, incorporating coupled heat and solute diffusion. Coarsening and remelting of the secondary arms were also simulated. They found that the base of the secondary arms did not decrease as predicted by coarsening theory, and the arms spacing remained unchanged when the arm grew into a steady state; however, the volume of the arms increased. Also, both remelting and coarsening of secondary arms were observed, which agreed with the coarsening theory and experimental observations. Theoretical and experimental models for predicting dendritic growth in multicomponent alloys are scarce due to the complexity of the task. One notable mathematical model was developed by Kirkwood, which considers the dissolution of small arms from their tips to determine secondary dendrite arm spacing (λ_2_) as a function of time during solidification for both isothermal and constant cooling rate conditions [18]. An existing expression used for calculating λ_2_ in binary alloys, based on dendrite ripening as the primary coarsening mechanism, was extended to multicomponent systems by Rappaz and Boettinger [19]. These researchers proposed a comprehensive model of equiaxed dendritic solidification for multicomponent alloys, capturing the interplay between dendrite growth kinetics and the global solute balance at the local grain scale. Their approach was validated against experimental λ_2_ values for various superalloys, demonstrating that the calculated values closely matched the experimental scatter.

Easton et al. [20] used a dendrite ripening model to predict secondary dendrite arm spacing (SDAS) for multicomponent aluminum alloys and validated their findings against experimental data. Their analysis revealed that the final SDAS was influenced by both the solidification time and the solute profile of the alloys. Intriguingly, despite the substantial variations in solidification times and solute segregation among the alloys, these two factors largely offset each other, resulting in surprisingly consistent SDAS predictions across the alloy range. The experimental and modeling results demonstrated that elements that induce significant constitutional undercooling near the onset of solidification, such as Ti, which significantly reduces grain size, have minimal impact on SDAS. Instead, elements with strong partitioning behavior towards the end of solidification were found to be more effective in suppressing SDAS coarsening. In Ref. [21], researchers extended an existing expression for calculating secondary dendrite arm spacing (λ_2_) of multicomponent alloys, which primarily considered dendrite ripening as the coarsening mechanism. This extension incorporated a back-diffusion treatment, broadening the range of non-equilibrium solidification conditions for which the predictions were valid. The proposed approach was validated against experimental λ_2_ values for ternary alloys cast both horizontally (H) and vertically (V) under transient heat flow conditions: Al-6 wt%Cu-4 wt%Si alloy (H and V), Al-3 wt%Cu-5.5 wt%Si alloy (H and V), Al-3 wt%Cu-9 wt%Si alloy (H), Al-6 wt%Cu-2.5/8 wt%Si alloys (H), and Al-3 wt%Cu-0.5 wt%Mg alloy (H). The predictions were found to be in good agreement with the experimental λ_2_ scatters for all the Al-Cu-Si alloys examined, with a slight tendency to overestimate the λ_2_ values for the only Al-Cu-Mg alloy cast. Sari et al. [22] proposed a model able to tackle the growth and the coarsening of secondary side dendrite arms with only two adjacent side arms in concurrence. Their model was applied to directional solidification of an Al-06 wt%Cu alloy in a Bridgman experiment. The authors reported a fast growth of both arms at an earlier stage of solidification, followed by remelting of the smaller arm. In addition, the numerical results were in good agreement with an available proposed time-dependent expression that covered growth and coarsening.

In previous publications, the authors proposed a method to predict the secondary dendrite arm spacing based on solidification parameters, such as temperature gradient, growth rate, and cooling rate. In the current work, a numerical model was developed to predict the evolution of secondary dendrite arm spacings during directional solidification of the Pb–Sn alloys. The analysis was based on the hypothesis that the secondary arm spacing is linearly related to the main dendrite tip radius. Therefore, a system of equations that correlated the thermal parameters with secondary dendrite arm spacing will be presented. The model was applied and compared to directional solidification experiments with different Pb–Sn alloys conducted by Çadirli et al. [23] that have been carried out to obtain data on dendritic growth under constant temperature gradients and different growth rates. In addition, the model presented in this paper was extended to consider dendrite growth incorporating curvature effects and solute diffusion. With these modifications, the model could be used to predict the coarsening phenomena for future research.

## 2. Hypothesis

**Figure 1:**
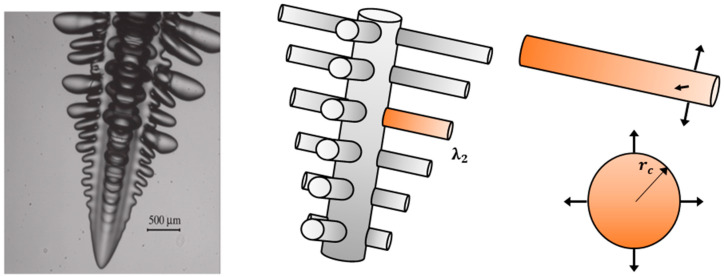
Schematic representation of the dendrite side branches which are simplified as cylinders. On the left, typical dendrite images were obtained from the high-resolution camera for uc = 0.2 K and initial acetone concentration C_0_ = 0.0086 mol% (almost pure SCN), (reprinted from Ref. [25], Copyright 2012, with permission from Elsevier). In the middle, simplifications of the side arms as cylinders. On the right, representation of one arm on both the front and side view.

The specific area of the solid/liquid interface is an important integral measure for the morphological evolution during the solidification process. In order to calculate the size of the side branches of the columnar dendrite, these side arms were simplified as cylinders (see Figure 1) which allowed us to use a mathematical model for these geometry types, [24,25]. An expression used for calculation of the arm radius, r_c_, of Pb–Sn alloys which is related to the calculation of dendrite arm growth, specifically considers the curvature effect as given by Gibbs–Thomson coefficient (Γ). In dendritic growth models, the dendrite arm radius (r_c_) evolves over time due to various factors such as diffusion, solute redistribution, and thermal gradients.

(1) $$\frac{dr_{c}}{dt}=\frac{D_{l}}{r_{c}ln\left(\frac{R_{f}}{r_{c}}\right)}\;\frac{C_{l}-C_{1}-\frac{\Gamma }{m_{l}\;r_{c}}}{C_{l}^{*}-C_{s}^{*}}$$

Equation (1) was proposed to calculate the growth rate which represented a rate equation governing the change in r_c_ with respect to time ($\frac{dr_{c}}{dt}$). It incorporated terms related to solute diffusion (D_l_), the dendrite arm radius (r_c_), a characteristic length scale (R_f_), concentration gradients (C_l_, C_1_, C_l_*, C_s_*), and parameters (Γ, m_l_) related to the system under consideration. The presence of the term ($D_{l}/r_{c}ln\left(R_{f}/r_{c}\right)$) suggested that solute diffusion plays a role in dendrite growth; the term (${(C}_{l}-C_{1}-\frac{\Gamma }{m_{l}r_{c}})/(C_{l}^{*}-C_{s}^{*})$) represented the concentration gradient driving dendrite growth, possibly incorporating solute partitioning effects and the influence of curvature, where, r_c_ on the right-hand side was calculated from the previous time step. This equation represented a simplified model for dendrite growth incorporating curvature effects and solute diffusion. Its derivation and specific application would depend on the underlying assumptions and context of the dendritic growth process being studied.

**Figure 2:**
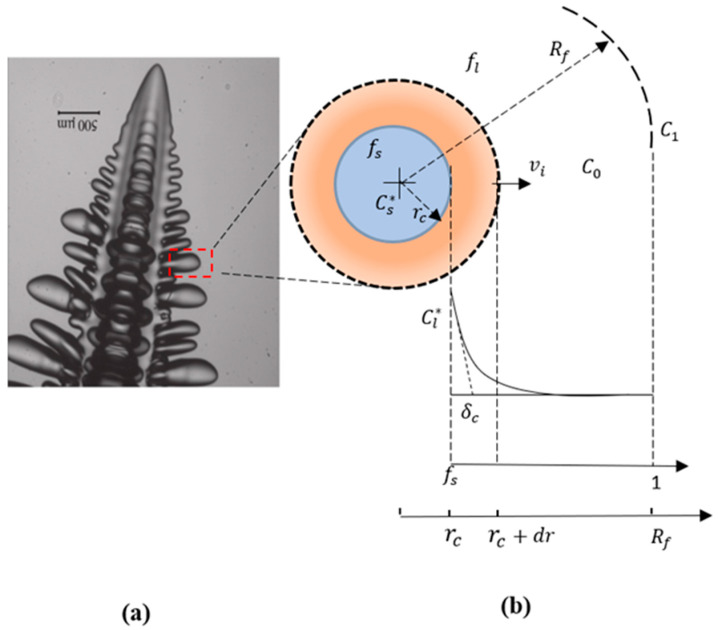
(**a**) Columnar dendrite (reprinted from Ref. [25], Copyright 2012, with permission from Elsevier). (**b**) Side view of a cylindrical arm enveloped in a cylindrical volume with a radius *R_f_*.

The arms had a radius, r_c_, that grew gradually with time according to Equation (2) as the solidification time proceeded inside a cylinder of radius R_f_ as shown in Figure 2. By multiplying Equation (1) by r_c_ and using ($r_{c}dr_{c}=\frac{1}{2}d{r_{c}}^{2}$), Equation (1) becomes as follows:

(2) $$\frac{d{r_{c}}^{2}}{dt}=2\frac{D_{l}}{ln\left(\frac{R_{f}}{r_{c}}\right)}\;\frac{C_{l}-C_{1}-\frac{\Gamma }{m_{l}\;r_{c}}}{C_{l}^{*}-C_{s}^{*}}$$

C_l_* is the solute concentration in liquid at the solid/liquid interface of the secondary arm, which is expressed by C_l_* = C_l_ − Γ/r_c_ where *C_l_* is the liquidus concentration taken from the phase diagram. C_s_*, is the solidus concentration at the equilibrium, Γ is the Gibbs–Thomson coefficient, and m_l_ is the liquidus slope.

This work was based on two main hypotheses. The first was to assume the initial secondary arm radius was equal to the tip radius of the main dendrite r_c_ = R_tip_. The second was that the envelope radius should be in the order of the diffusion length at the tip R_f_ ~2D_l_/v_tip_, v_tip_ being the tip growth velocity.

Inside this envelope, the solid fraction, f_s_, of the arm growth was defined as the ratio of the volume of the growing arm to the volume of the total envelope with radius *R_f_*. It developed in accordance with Equation (3) from a finite value (when r_c_ = R_tip_) to possibly reach f_s_ = 1 (when r_c_ = R_f_).

(3) $$f_{s}=\frac{V_{r}}{V_{tot}}=\left(\frac{\pi {r_{c}}^{2}L}{\pi R_{f}^{2}L}\right)=\frac{{r_{c}}^{2}}{R_{f}^{2}},\;\mathrm{with}\;f_{l}=1-f_{s}$$

The solute distribution field was assumed to be isotropic around each secondary arm as shown in Figure 2. The average liquid concentration, C_avr_, inside the envelope was obtained by solving the conservation equation of the solute at equilibrium and at the solid–liquid interface as given in Equation (4).

(4) $$\frac{\partial {(f_{l}\;C}_{avr})}{\partial t}=-\frac{\partial \left(f_{s}\right)}{\partial t}\left(C_{s}^{*}-C_{l}^{*}\right)$$

The concentration at *R_f_* is noted by, C_1_, v_r_ is the growth rate of the arm, and δc is the diffusion distance that can be written as: δ_c_ = D_l_/v_r_, where *D_l_* is the liquid diffusion.

The left term of Equation (4) represented the rate change of the average concentration (C_avr_) of the liquid phase with respect to time. However, the term on the right-hand side represented the rate of change of the solid fraction with respect to time. The equation essentially described the change in the average concentration of the liquid phase over time as a result of the solidification process. It related this change to the rate of change of the solid fraction and the concentration difference between the solid and liquid phases at equilibrium. This type of equation is commonly encountered in models describing phase transformations, where the evolution of different phases (solid, liquid) and their associated properties (e.g., concentration) are considered over time during solidification processes.

In the cylindrical coordinate, the diffusion equation [26] can be written in the form:

(5) $$\frac{\partial C}{\partial t}=D_{l}\left(\frac{\partial^{2}C}{\partial r^{2}}+\frac{1}{r}\frac{\partial C}{\partial r}\right)$$

Since the growth of secondary arms is very slow, the steady state condition can be assumed ($\frac{\partial C}{\partial t}=0$), [27]. In this condition, Crank [28] presented the solution of Equation (5) according to the following form:

(6) $$C=aln\left(r_{c}\right)+b$$

where a and b are constants to be determined from the boundary conditions:

$$\left\{\begin{array}{c} C=C_{1}\;at\;r_{c}=R_{f}\\ C=C_{l}^{*}\;at\;r_{c}=r \end{array}\right.$$

Equation (6) gives an infinite concentration at $r_{c}$ = *∞*. So, the boundary condition must be limited at some radius noted *R_f_*. It represents the available space around the arm. This radius corresponds to the radius at which the tip Peclet number equals 1. For a specific liquid diffusion (D_l_) and initial dendrite tip velocity (v_tip_), R_f_ is assumed for now to be equal to:

(7) $$R_{f}=2\frac{D_{l}}{v_{tip}}$$

After the application of the boundary conditions, the solution becomes:

(8) $$C\left(r\right)=\frac{C_{1}\ln\left(\frac{r}{r_{c}}\right)+C_{l}^{*}\ln\left(\frac{R_{f}}{r}\right)}{\ln\left(\frac{R_{f}}{r_{c}}\right)\;}$$

The general form of Equation (8) enables the estimation of concentration during the growth.

The average liquid concentration (C_avr_) can also be estimated as a function of the liquid concentration (C_1_) at R_f_, as given in Equations (9) and (10).

(9) $$C_{avr}=\frac{1}{\pi \left(R_{f}^{2}-r_{c}^{2}\right)}\int_{r_{c}}^{R_{f}}C\left(r\right)\cdot 2\pi rdr$$

Then:

(10) $$C_{avr}=\frac{1}{\left(R_{f}^{2}-r_{c}^{2}\right)\ln\left(\frac{R_{f}}{r_{c}}\right)}\left[\begin{array}{c} C_{1}\left(R_{f}^{2}\ln\left(R_{f}\right)-r_{c}^{2}\ln\left(r_{c}\right)-\left(\frac{1}{2}+\ln\left(r_{c}\right)\right)\left(R_{f}^{2}-r_{c}^{2}\right)\right)+\\ C_{l}^{*}\left(\left(\frac{1}{2}+ln\left(R_{f}\right)\right)\left(R_{f}^{2}-r_{c}^{2}\right)-R_{f}^{2}\ln\left(R_{f}\right)+r_{c}^{2}\ln\left(r_{c}\right)\right) \end{array}\right]$$

As a solid/liquid interface advances with growth rate (v_i_), the liquid concentration (C_l_*), and the solid concentration (C_s_*), the quantity of the solute rejected per unity time will be v_i_(C_l_* − C_s_*); this must be balanced by the creation of a concentration gradient in the liquid, thus [23],

(11) $$v_{i}\left(c_{l}^{*}-c_{s}^{*}\right)=\frac{D_{l}}{r_{c}ln\left(\frac{R_{f}}{r_{c}}\right)}\left(C^{*}-C_{1}\right)$$

where $v_{i}=\frac{dr_{c}}{dt}$ and $C^{*}=C_{l}-\frac{\Gamma }{m_{l}r_{c}}$.

The “far field” liquid concentration C_1_ at the position R_f_ can be estimated from Equation (9), [24].

(12) $$C_{1}=\frac{\left(R_{f}^{2}-r_{c}^{2}\right)\ln\left(\frac{R_{f}}{r_{c}}\right)C_{avr}-C_{l}^{*}\left(\left(\frac{1}{2}+ln\left(R_{f}\right)\right)\left(R_{f}^{2}-r_{c}^{2}\right)-R_{f}^{2}\ln\left(R_{f}\right)+r_{c}^{2}\ln\left(r_{c}\right)\right)}{\left(R_{f}^{2}\ln\left(R_{f}\right)-r_{c}^{2}\ln\left(r_{c}\right)-\left(\frac{1}{2}+\ln\left(r_{c}\right)\right)\left(R_{f}^{2}-r_{c}^{2}\right)\right)}$$

Thus, the main equation systems were Equations (2), (4), and (12) that predict the evolution of arm radius ($r_{c}$), average liquid concentration (C_avr_), and the concentration (C_1_) at (*R_f_*).

## 3. Main Dendrite Tip Kinetic Growth

**Figure 3:**
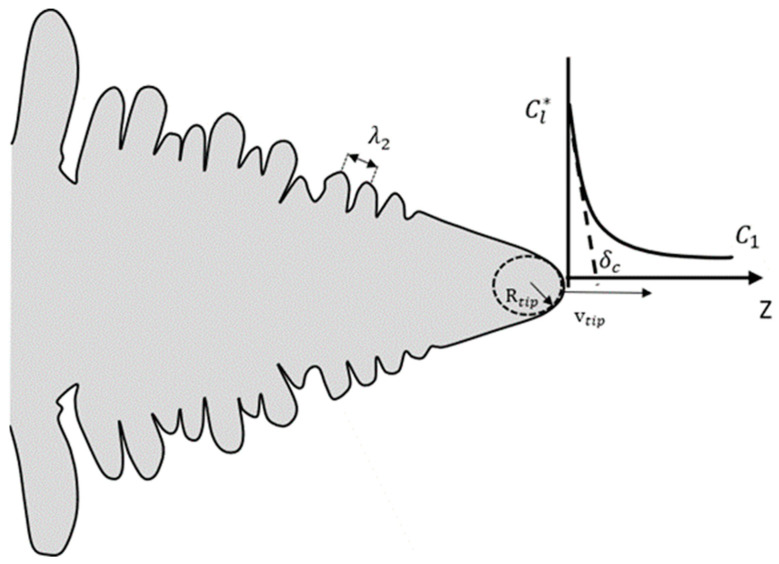
Schematic of dendrite tip having a paraboloid evolution shape and the solute distribution ahead of the dendrite tip.

To obtain a value of the radius R_f_ it was necessary to estimate the main dendrite tip growth velocity. The growth kinetics of a dendrite are strongly dependent on the mass transfer that occurs near its tip. Figure 3 shows a schematic of the dendrite composition ahead of the tip dendrite along its z-axis. Note that the composition at the tip is C_l_* and declines to some value C_1_ at distance δ_c_ (the diffusion distance of the solute) ahead of the tip [29]. The change in the melting point is due to the curvature effect ΔT_c_ often called curvature or Gibbs–Thomson undercooling. The resolution of these phenomena is based on the Ivantsov function which can treat those phenomena that occur ahead of this paraboloid shape.

In order to treat those phenomena that occur at the dendrite tip that have a paraboloid of revolution shape, Ivantsov gave a mathematical solution for this shape by defining a relation between the supersaturation (Ω) and the Ivantsov function (I_v_ (Pe)) as given in Equations (13) and (14), respectively, where E_1_ is the exponential integral function given elsewhere [30]. An approximation was suggested by Kurz [26] in order to calculate the tip Peclet number by inversing the Ivantsov solution as defined in Equation (15).

(13) $$Ω=I_{v}\left(Pe\right)=\frac{C_{l}^{*}-C_{0}}{C_{l}^{*}\;\left(1-k\right)}$$

(14) $$I_{v}\left(Pe\right)=Pe\exp\left(Pe\right){\cdot E}_{1}\left(Pe\right)$$

(15) $${Pe}_{1}=-\frac{Ω}{\ln\left(Ω\right)}$$

(16) $$Error=\frac{Pe}{{Pe}_{1}}$$

(17) $$Pe\left(Ω\right)=I_{v}^{-1}\left(Ω\right)=-\frac{Ω}{ln\left(Ω\right)}F(Ω)$$

**Figure 4:**
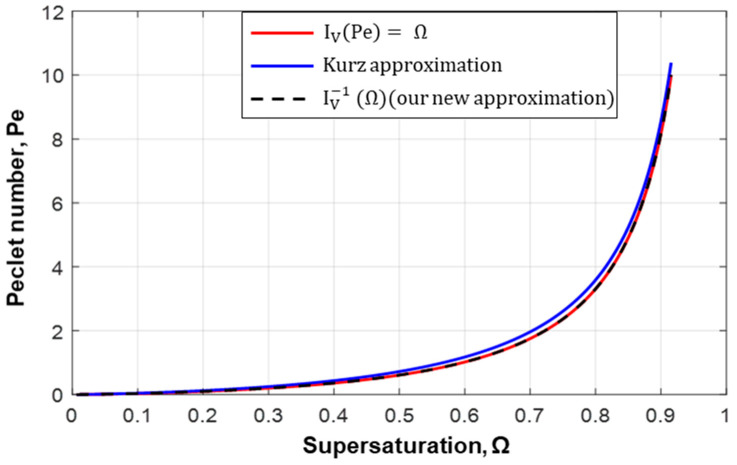
The supersaturation (Ω) vs. Peclet number given by the Ivantsov function (Equation (14)) presented by red, the Kurz approximation (Equation (15)) presented by blue, and our new approximation presented by the black dashed line (Equation (17)).

Figure 4 illustrates the variation of Peclet number as a function of the supersaturation, Ω, that was given by both the Ivantsov function and Kurz approximation. The estimated Peclet numbers from the Ivantsov function and Kurz approximation were very close to each other. The accuracy of the Kurz approximation for estimating *Pe* with respect to the Ivantsov function showed a relative error of about 20% at small supersaturation values as presented in Figure A2 in Appendix B which is relatively high and it cannot be negligible. However, the deviation decreased at higher supersaturation values. In order to obtain a more accurate approximation we proposed a new solution (Equation (17)), where (*I_v_*^−1^ (Ω)) was the inverse Ivantsov solution (new proposed approximation) and F(Ω) was a fitting function of the error equation (more detailed in Appendix B). In Figure 4, one can easily notice that the new proposed approximation was in much better agreement with the Ivantsov profile.

In light of the previous approximation that allowed us to calculate the Peclet number for a given supersaturation (Ω), the corresponding tip radius (R_tip_) could be calculated according to Kharicha et al. [31] by Equation (18), where σ is the stability constant (σ = 1/4п^2^), Pe is the Peclet number that is obtained from Equation (17), and C_l_* and C_s_* are the liquidus and solidus concentration, respectively. Therefore, the corresponding tip velocity (v_tip_) could be evaluated based on the formula of the Peclet number (Pe = R_tip_ v_tip_/2D_l_), as defined in Equation (19).

(18) $$\;R_{tip}=\frac{\Gamma }{2\;Pe\;m_{l}\;\sigma \;(C_{l}^{*}-C_{s}^{*})}$$

(19) $$v_{tip}=\frac{2\;Pe\;D_{l}}{R_{tip}}$$

## 4. Comparison of the Solidification Parameters between Experiment Measurement and Numerical Predictions

The present model was then tested against the experiments presented by Çadirli et al. [23]. They studied the effect of several parameters (growth rate *V*, temperature gradient DT, and cooling rate CR) during directional solidification of different Pb–Sn binary alloys on the formed primary and secondary dendrite arm spacings $\lambda_{1}$ and $\lambda_{2}$, respectively. The validation of the above equations system concerned the prediction of both the secondary dendrite arm spacing and tip velocity. To achieve that, a numerical code was created in the trial to reproduce the experiments. The liquidus slope (m_l_) of the corresponding phase diagram is defined in Equation (20), where, the temperature (T) is a function of time (T(t) = T_L_ − CR × t) and CR is the cooling rate defined in Equation (21). The liquidus and solidus concentrations (*C_l_* and *C_s_*) of the Pb–Sn phase diagram are presented in Appendix A.

(20) $$m_{l}\left(T\right)=\frac{1}{\left(\frac{\partial C_{l}}{\partial T}\right)}=\frac{1}{{{4p}_{1}T}^{3}+{{3p}_{2}T}^{2}+2p_{3}T+p_{4}}$$

where *p*_1–4_ are constants presented in Table A1 in Appendix A.

The cooling rate used in these experiments was calculated as a function of the thermal gradient DT (measured in (°C/m)) multiplied by the tip velocity v_tip_, as expressed in Equation (21). The final radius *R_f_* was expressed as a function of Peclet number pondered with the corrective factor (A = 1, 0.5 or 0.3) and tip radius (R_tip_) as defined in Equation (22) that could predict the value of the secondary dendrite arm spacing.

(21) $$CR=DT\;v_{tip}[{}^{\circ}C/s]$$

(22) $$R_{f}=A\frac{1}{{2P}_{e}}R_{tip}$$

Based on experimental data, Hachani et al. [32] have provided the following estimation of the diffusion coefficient for Pb in the liquid alloy as a function of temperature:

(23) $$D_{l}\left(T\right)=1.4\times 10^{-7}\exp\left(-\frac{2.29\times 10^{4}}{RT}\right)\left[m^{2}/s\right]$$

where 1.4 × 10^−7^ is a constant prefactor, 2.29 × 10^4^ represents an activation energy term in units of Joules per mole (J/mol), R is the ideal gas constant (R = 8.314 J/mol/K), and T is the temperature in Kelvin.

The numerical and the experimental measurements are reported in Table 1. The numerical results were obtained for fixed thermal gradient *DT*, and by adapting the initial undercooling of each Pb–Sn alloy that achieved the same tip velocities in the simulation and experiments. Once this condition was achieved, the corresponding parameters of this growth velocity were presented as shown in Table 1. Also, the cooling rate could be calculated by Equation (21). In addition, the corrective factor, *A*, was chosen to slightly modify the envelope radius R_f_.

**Figure 5:**
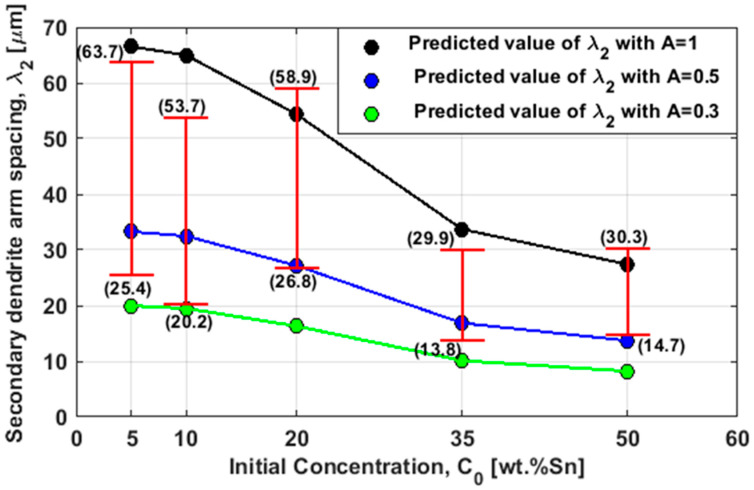
The secondary dendrite arm spacing ***λ***_2_ versus the initial concentration C_0_. The red margins represent the minimum and maximum ***λ***_2_ of the experiments [23]. While the red vertical lines are the ***λ***_2_ ranges of each alloy. The black, blue, and green heads are the computed ***λ***_2_ value obtained with A = 1, 0.5, and 0.3, respectively.

Figure 5 presents the secondary dendrite arm spacing versus the initial concentration of the five studied alloys. All the experimental results were found to lie between the black and the green lines obtained with A = 1 and A = 0.5; few data needed a lower A down to 0.3. Based on our theory, the results obtained with A = 1 represented the fully developed arms. Lower values obtained with A = 0.3–0.5 did not finish their growth or were stopped due to competition in growth between parallel arms. In addition, the predicted SDAS values decreased as the initial concentration increased (higher value of SDAS was obtained with the smaller initial concentration 5 wt%Sn and lower SDAS with 50 wt%Sn).

**Figure A1:**
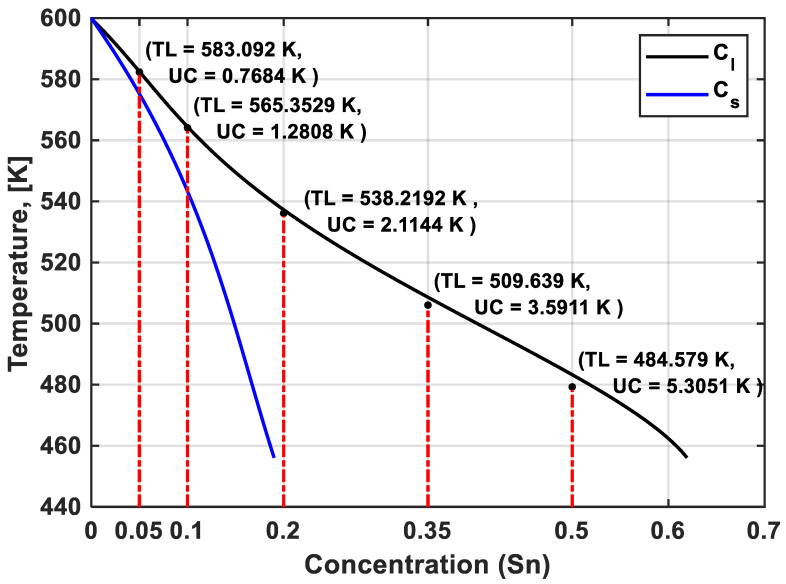
Phase diagram of Pb-Sn (right part), liquidus and solidus concentration are presented in black and blue. Also, the blackheads correspond to initial arm undercooling (at t = 0 s) as shown in Table A1. While, the red dashed lines represent the initial concentration of the studied alloys.

**Figure A2:**
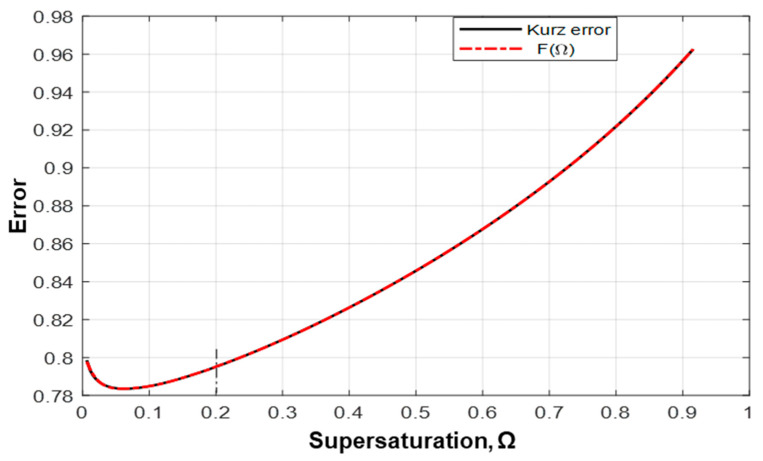
Kurz error equation (black line) and the fitting function (F(Ω), dashed red line) for various Ω.

On the other hand, a very good agreement between the numerical and the experimental velocities was obtained as reported in Table 1. A very close agreement was achieved for Pb-5 wt%Sn as given in Table 1, where the experimental velocity was 18.58 µm/s and the numerical predicted velocity was 18.59 µm/s. However, for Pb-10 wt%Sn, Pb-20 wt%Sn, Pb-35 wt%Sn, and Pb-50 wt%Sn, the numerical and the experimental tip velocities were exactly the same; their values were 16.36, 15.29, 18.45, and 17.11 µm/s, respectively. In addition, the initial undercooling for different Pb–Sn alloys is also presented in Table 1 and also presented in Appendix A by the blackheads. Based on the results presented in Table 1 and in Figure A1 (in Appendix A), it can be easily noticed that the tip undercooling of the Pb-5 wt%Sn alloy was 0.7684 °C. Then, as the initial concentration of the alloy (C_0_) increased from 5-wt% to 50-wt% (first column in Table 1), the tip undercooling increased from 0.7684 K to 5.3051 K. In addition, faster cooling rates (CR = 0.037 °C/s) were obtained for higher tip undercoolings and higher initial concentrations (C_0_ = 50 wt%). Moreover, a maximum tip Peclet number of about 0.08 was obtained with the Pb-5 wt%Sn alloy which corresponded to Ω = 0.1 (see Table 1). The corresponding error of the Kurz approximation for Ω ~ 0.1 was about 20% (see Figure A2 in Appendix B) which is relatively high and is not negligible. Our proposed correction (Equation (17)) provided us with a better estimation of the tip Peclet number. In our model, the accuracy of the secondary dendrite arm spacing prediction was directly related to the knowledge of tip kinetics.

## 5. Conclusions

The present paper deals with the prediction of secondary dendrite arm spacing during directional solidification of different Pb–Sn alloys. The model showed its effectiveness to predict SDAS based solely on tip supersaturation and cooling rate. The numerical results were obtained for the fixed thermal gradient DT, and by adapting the initial undercooling of each Pb–Sn alloy that achieved tip velocities similar to those of other experiments. In good qualitative agreement with other experiments, the model predicted formation of coarser arms for lower initial alloy concentrations, slower cooling rates, and the values of the SDAS decreased as the initial concentration increased. Quantitatively, the calculated SDAS and tip velocities by the proposed model exhibited a very good agreement with the available measurements. Further model verifications in the future will aimed at considering other alloy systems. However, sufficient thermophysical properties, dendrite tip, and experimental conditions need to be accurately known as for the Pb–Sn alloys used in this work. This model will be implemented as a sub-grid model for the prediction of the evolution of the main secondary arms spacing during microscopic solidification processes which are important for freckles prediction and to control the mushy zone permeability.

## Figures and Tables

**Table 1:** Comparison between experimental and numerical solidification parameters of the Pb–Sn alloy.

50%			35%			20%			10%			5%			**C_0_**	
17.11			18.45			15.29			16.36			18.58			**Tip Velocity** **(m/s)** **×10^−6^**	**Experimental**
0.037			0.031			0.0254			0.0237			0.016			**CR** **(°C/s)**	
14.7–30.3			13.8–29.9			26.8–58.9			20.2–53.7			25.4–63.7			***λ*_2_** **(m)** **×10^−6^**	
5.3051			3.5911			2.1144			1.2808			0.7684			**Under** **Cooling** **(K)**	
17.11			18.45			15.29			16.36			18.59			**Tip Velocity** **(m/s)** **×10^−6^**	**Numerical calculation**
0.3	0.5	1	0.3	0.5	1	0.3	0.5	1	0.3	0.5	1	0.3	0.5	1	**A**	
08.22	13.71	27.41	10.11	16.86	33.71	16.30	27.17	54.35	19.49	32.48	64.96	19.97	33.28	66.5	***λ*_2_ predicted** **(m)** **×10^−6^**	
0.0825			0.0932			0.0964			0.1158			0.1507			**Ω**	
0.0331			0.0393			0.0412			0.0537			0.0797			**Pe**	
1.81			2.65			4.48			6.98			10.57			**R_tip_** **(m)** **×10^−6^**	
0.469			0.622			0.831			1.063			1.237			**D** **(m^2^/s)** **×10^−9^** [32]	

## Data Availability

Data are contained within the article.

## Abbreviations

$C_{0}$	The initial concentration of the alloy	T	Temperature, (K)
C_1_	Liquid concentration at r = R_f_	T_L_	Liquidus temperature, (K)
C_avr_	Average liquid concentration	DT	Thermal gradient, (K/m)
C_l_*, C_s_*	Liquidus and solidus concentrations	Γ	Gibbs Thomson coefficient, (m K)
m_l_	Liquidus slope, (K wt%^−1^)	r_c_	The arm radius, (m)
D_l_	Liquid diffusion coefficient, (m^2^ s^−1^)	R_f_	The radius of the cylindrical envelope, (m)
Ω	Supersaturation	R_tip_	The radius of the tip dendrite, (m)
UC	Initial undercooling, (K)	V, v_tip_	Experimental and numerical growth rate of the arm, (m/s)
CR	Cooling rate, (K s^−1^)	σ	The stability constant (σ = 1/4п^2^)
δ_c_	Diffusion distance, (m)	f_l_, f_s_	Liquid and solid fractions
E_1_	The integral exponential function	A	Corrective factor
I_v_	Ivantsov function	λ _1_, λ_2_	Primary and secondary dendrite arm spacing, (m)
Pe	Peclet number	V_r_	Volume of the arm, (m^3^)
p_i_,q_i_	constants	V_tot_	Total envelope volume, (m^3^)
R	Ideal gas constant, (8.314 J/mol/K)	F	Fitting function

## Appendix A

(A1)
$$C_{l}(T)=p_{1}T^{4}+p_{2}T^{3}+p_{3}T^{2}+p_{4}T+p_{5}$$

(A2)
$$C_{s}(T)=q_{1}T^{4}+q_{2}T^{3}+q_{3}T^{2}+q_{4}T+q_{5}$$

**Table A1:** The corresponding constants of the liquidus and solidus concentration of the Pb–Sn phase diagram.

C_l_ (T)				
$p_{1}$	$p_{2}$	$p_{3}$	$p_{4}$	$p_{5}$
−2.9739 × 10^−9^	6.4158 × 10^−6^	−5.1601 × 10^−3^	1.8292	−240.28
		C_s_ (T)		
$q_{1}$	$q_{2}$	$q_{3}$	$q_{4}$	$q_{5}$
8.5229 × 10^−11^	−2.0961 × 10^−7^	1.8436 × 10^−4^	−7.0726 × 10^−2^	10.296

## Appendix B

The fitting function of the error F(Ω) was separated into two parts (as separated by black dashed line at Ω = 0.2 in Figure A2), the first one (Equation (A3)) is for Ω < 0.2 and the second one for Ω > 0.2 defined in Equation (A4).

(A3) $$F\left(Ω\right)=p_{1}{Ω}^{6}+p_{2}{Ω}^{5}+{p_{3}Ω}^{4}+{p_{4}Ω}^{3}+{p_{5}Ω}^{2}+p_{6}Ω+p_{7}$$

(A4) $$F(Ω)={q_{1}Ω}^{4}+{q_{2}Ω}^{3}+{q_{3}Ω}^{2}+q_{4}Ω+q_{5}$$

where $p_{1}$ − $p_{7}$ and $q_{1}$ − $q_{5}$ are constants presented in Table A2.

**Table A2:** The corresponding constants of the fitting function F(Ω).

F(Ω) (Equation (A3))						
$p_{1}$	$p_{2}$	$p_{3}$	$p_{4}$	$p_{5}$	$p_{6}$	$p_{7}$
11,460	−8248.5	2375.8	−351.2	28.753	−1.2251	0.80449
		F(Ω) (Equation (A4))				
$q_{1}$	$q_{2}$	$q_{3}$	$q_{4}$	$q_{5}$		
0.19968	−0.34927	0.34839	0.020202	0.77974		
